# Mode of Delivery and Neonatal Outcomes of Preterm Deliveries: A Retrospective Study in Greece

**DOI:** 10.3390/medicina60010010

**Published:** 2023-12-20

**Authors:** Kyriaki Mitta, Ioannis Tsakiridis, Georgios Kapetanios, Antigoni Pavlaki, Efthymios Tarnanidis, Themistoklis Dagklis, Apostolos Athanasiadis, Apostolos Mamopoulos

**Affiliations:** 1 Third Department of Obstetrics and Gynecology, School of Medicine, Faculty of Health Sciences, Aristotle University of Thessaloniki, 541 24 Thessaloniki, Greece; kmittb@auth.gr (K.M.); kapetaniosg@hotmail.gr (G.K.); tarnanidis_mimis@hotmail.com (E.T.); tdagklis@auth.gr (T.D.); apathana@auth.gr (A.A.); amamop@auth.gr (A.M.); 2 Neonatal Intensive Care Unit, Hippokrateio General Hospital of Thessaloniki, 541 24 Thessaloniki, Greece; antigonipavlaki@yahoo.gr

**Keywords:** preterm birth, outcome, mode of delivery, vaginal, cesarean, neonatal death, respiratory disorders, necrotizing enterocolitis, intraventricular hemorrhage

## Abstract

*Background and Objectives*: Preterm birth is a significant concern in obstetrics and neonatology since preterm neonates are at higher risk of various health complications and may require specialized care. The optimal mode of delivery in preterm birth is a matter of debate. This study aimed to evaluate the mode of delivery in preterm neonates and the associated neonatal outcomes. *Material and Methods*: This was a retrospective cohort study including all preterm neonates born between January 2010 and December 2020 at the 3rd Department of Obstetrics & Gynecology of Aristotle University of Thessaloniki, Greece. The mode of delivery in relation to gestational age groups and the cause of preterm birth were analyzed. Neonatal outcomes were also evaluated according to gestational age, indication and mode of delivery. *Results*: A total of 1167 preterm neonates were included in the study; the majority of them were delivered via cesarean section (76.1%). Most of the preterm neonates (*n* = 715; 61.3%) were delivered at 32^+0^–36^+6^ weeks, while cesarean section was the most common mode of delivery after 28^+0^ weeks. Furthermore, spontaneous onset of labor (OR: 6.038; 95% CI: 3.163–11.527; *p* < 0.001), multiple gestation (OR: 1.782; 95% CI: 1.165–2.227; *p* = 0.008) and fetal distress (OR: 5.326; 95% CI: 2.796–10.144; *p* < 0.001) were the main causes of preterm delivery at 32^+0^–36^+6^ weeks. The overall mortality rate was 8.1% among premature neonates. Regarding morbidity, 919 (78.7%) neonates were diagnosed with respiratory disorders, 129 (11.1%) with intraventricular hemorrhage and 30 (2.6%) with necrotizing enterocolitis. Early gestational age at delivery was the main risk factor of neonatal morbidity and mortality. Notably, the mode of delivery did not have any impact on neonatal survival (OR: 1.317; 95% CI: 0.759–2.284; *p* = 0.328), but preterm neonates born via cesarean section were at higher risk of respiratory disorders, compared to those born via vaginal delivery (OR: 2.208; 95% CI: 1.574–3.097; *p* < 0.001). *Conclusions*: Most preterm deliveries occurred in the moderate-to-late preterm period via cesarean section. Early gestational age at delivery was the main prognostic factor of neonatal morbidity and mortality, while the mode of delivery did not have any impact on neonatal survival. Future research on the mode of delivery of the preterm neonates is warranted to establish definitive answers for each particular gestational age.

## 1. Introduction

In Europe, preterm birth is defined as delivery occurring between 22^+0^ and 37^+0^ weeks of gestation; its prevalence ranges from 5 to 18%, with only 0.3–0.5% occurring before the 28th week of gestation [1,2,3]. The main risk factors for preterm birth are the use of assisted reproductive technology; multiple pregnancies; previous history of preterm birth; surgery on the cervix; preterm prelabor rupture of the membranes; tobacco, alcohol, or drug use; consumption of sugary beverages in large quantities; and a Western-style diet [4,5,6,7]. Other factors related to preterm birth include unemployment, chronic stress and family status [8,9]. The risk of preterm birth is associated with the mother’s demographic characteristics; it varies according to race and ethnicity and is associated with increased maternal age [6].

The optimal mode of delivery for preterm neonates still remains controversial; the option of performing elective cesarean section was suggested to reduce incidents of hypoxia that may occur during a preterm birth, but this theory was not evidence-based [10]. A Cochrane review on the mode of delivery in preterm singleton pregnancies found that the frequency of birth trauma, asphyxia and perinatal mortality is the same for cesarean and vaginal delivery [11]. Moreover, maternal mortality associated with vaginal delivery in preterm pregnancies is significantly less compared to cesarean section; vaginal birth should probably be chosen for preterm neonates when there are no obstetric reasons for performing a cesarean section [11,12]. However, several studies suggest that in singleton pregnancies delivering preterm with cephalic presentation and an estimated fetal weight below 1500 g, cesarean delivery reduces neonatal mortality [13,14] and is associated with better neonatal outcomes in neonates born between the 22nd and 25th weeks of gestation [15,16].

This study aimed to evaluate the mode of delivery in preterm neonates and also evaluate the associated neonatal outcomes.

## 2. Material and Methods

This was a retrospective cohort study including all preterm neonates born between January 2010 and December 2020 at the 3rd Department of Obstetrics and Gynecology of the Aristotle University of Thessaloniki, Greece. This unit is a tertiary referral center for a large area in Northern Greece. All the neonates born prematurely were included regardless of their mode of delivery and whether they were admitted to the neonatal intensive care unit. As per national guidance, prematurity was defined as delivery between 24^+0^ and 36^+6^ weeks of gestation; preterm neonates were further classified in three groups according to gestational age at delivery: 24^+0^–27^+6^ weeks (extremely preterm), 28^+0^–31^+6^ weeks (early preterm) and 32^+0^–36^+6^ weeks of gestation (moderate-to-late preterm). In Greece, an increase in preterm births has been observed in recent years, coinciding with a concurrent decline in overall birth rates. The mean annual delivery rate in our department was about 1500 births per year.

To date, since no relevant guidelines exist, the mode of delivery is chosen by the attending physician. As a local policy, all non-cephalic fetuses, twin pregnancies and growth-restricted fetuses at <32 weeks are delivered by cesarean section unless they present at the active phase of labor with a cephalic presentation. Moreover, as per national and international guidelines, all pregnant women < 34 weeks received corticosteroids in anticipated preterm delivery within one week [17]. Moreover, magnesium sulphate was administered in pregnant women < 32 weeks in imminent preterm delivery [17]. In cases of preterm prelabor rupture of membranes (PPROM), expectant inpatient management up to 34 weeks was offered (including antibiotics for 7 days). Preeclampsia was addressed with antihypertensive medications for up to 37 weeks, taking into account the severity of symptoms and blood pressure control. For cases with spontaneous onset of labor, corticosteroids and tocolytic therapy for 48 h were administered up to 34 weeks. In cases of fetal growth restriction (FGR), an individualized approach, considering growth percentile and Doppler findings, was applied to determine the timing of delivery. The majority of cases were managed as outpatients by maternal–fetal medicine specialists, except those complicated by PPROM, HELLP syndrome and severe preeclampsia non-responsive to oral antihypertensive drugs.

The mode of delivery was analyzed based on gestational age at delivery, while the cause leading to prematurity was also investigated, and a comparison was performed between the causes and the gestational age. Furthermore, a comparison between the causes of delivery and the type of delivery for these neonates was performed as well. The neonatal outcomes, specifically mortality, morbidity, respiratory disorders (including transient tachypnea, neonatal respiratory distress syndrome, bronchopulmonary dysplasia, meconium aspiration syndrome, persistent pulmonary hypertension, pneumonia and apnea), intraventricular hemorrhage (IVH) and necrotizing enterocolitis (NEC) were evaluated in relation to gestational age, mode of delivery and causes of prematurity.

The women consented to the anonymity of their data and their use for research purposes, while no incentives were provided. Following the policy for observational studies that do not involve any intervention or modification to the routine care of the patients, no institutional board review was required for this study [18]. Qualitative variables were presented as *n* (%). Univariate analyses between categorical variables were conducted using the Chi-square test. Subsequently, multivariate analyses were performed using logistic regression models to identify independent risk factors for the studied neonatal complications (backward model). The *p*-value was calculated as two-tailed, and results were considered statistically significant if *p* < 0.05. The statistical analysis was carried out using the SPSS 28.0 statistical software package (IBM, Armonk, NY, USA).

## 3. Results

A total of 1167 preterm neonates were born during the study period and were included. The analysis of the data revealed that 127 neonates (10.9%) were born between 24^+0^ and 27^+6^ weeks, 325 (27.8%) between 28^+0^ and 31^+6^ weeks and 715 (61.3%) were born between 32^+0^ and 36^+6^ weeks of gestation. Furthermore, 279 (23.9%) neonates were born vaginally, whereas 888 (76.1%) were born via cesarean section; during this period, the mean cesarean section rate of all deliveries (including the preterm ones) was 48%. Taking into consideration that the total number of births during the study period was 15,450, the rate of births before 32^+0^ weeks was 2.9% and after 32 weeks 4.6%, whereas the rate of all preterm births (<37 weeks) was 7.6%.

Before 28^+0^ weeks of gestation, the odds of cesarean section were lower (OR: 0.260; 95% CI: 0.176–0.384; *p* < 0.001), whereas at 28^+0^–31^+6^ weeks, there was an increased chance of cesarean section compared to 32^+0^–36^+6^ weeks (OR: 1.561; 95% CI: 1.104–2.208; *p* = 0.012) (Table 1). Regarding the causes of prematurity, in 577 (49.4%) cases, there was a spontaneous onset of labor (contractions); in 219 (18.8%) cases, there was PPROM; while in 128 (11%) cases, preeclampsia or HELLP syndrome was diagnosed. FGR was diagnosed in 191 (16.4%) preterm neonates and 374 (32%) were twins or triplets (179 twins and 8 triplet pregnancies). Non-cephalic fetal presentation appeared in 104 (8.9%) fetuses and 265 (22.7%) were complicated with fetal distress. Following multivariate analysis, we found that cesarean section was more likely to be performed in preterm cases not being in labor at delivery (OR: 75.440; 95% CI: 16.654–341.731; *p* < 0.001), in multiple gestations (OR: 11.002; 95% CI: 6.806–17.785; *p* < 0.001), in fetuses at non-cephalic presentation (OR: 137.230; 95% CI: 18.694–1.007.394; *p* < 0.001) and in cases complicated by fetal distress (OR: 8.178; 95% CI: 3.398–19.686; *p* < 0.001) (Table 2).

We performed a subgroup analysis to assess the causes of prematurity according to different gestational age groups; spontaneous onset of labor (OR: 6.038; 95% CI: 3.163–11.527; *p* < 0.001), multiple gestation (OR: 1.782; 95% CI: 1.165–2.227; *p* = 0.008) and fetal distress (OR: 5.326; 95% CI: 2.796–10.144; *p* < 0.001) were more common at 32^+0^–36^+6^ weeks than at 24^+0^–27^+6^ weeks of gestation. Moreover, preterm delivery due to severe FGR was less common at 28^+0^–31^+6^ weeks than at 24^+0^–27^+6^ weeks (OR: 0.142; 95% CI: 0.047–0.430; *p* < 0.001) (Table 3).

Further subgroup analyses were performed evaluating the mode of delivery for each gestational age group according to different causes of preterm delivery; it was found that no labor was associated with an increased chance of cesarean delivery in all gestational age groups. For cases complicated by PPROM, there was an increased risk for cesarean section at 24^+0^–27^+6^ weeks (OR: 3.094; 95% CI: 1.105–8.668; *p* = 0.032). In cases of severe preeclampsia or HELLP syndrome, there was a significantly increased risk for cesarean section in all gestational age groups. FGR and multiple gestation were risk factors for cesarean delivery after 28^+0^ weeks of gestation. Moreover, non-cephalic fetal presentation and fetal distress were risk factors for cesarean section in all groups (Supplementary Tables S1–S7).

Regarding neonatal outcomes, 919 (78.7%) preterm neonates were diagnosed with respiratory disorders, 129 (11.1%) with IVH, 30 (2.6%) with NEC and 95 (8.1%) did not survive. With regard to neonatal survival, the mode of delivery was not found to be a risk factor for neonatal mortality, whereas early gestational age at delivery was associated with an increased risk for neonatal death. Specifically, neonatal death was almost 129 times higher at 24^+0^–27^+6^ weeks compared to 32^+0^–36^+6^ weeks (OR: 129.493; 95% CI: 49.761–336.979; *p* < 0.001) and 13 times higher at 28^+0^–31^+6^ weeks compared to 32^+0^–36^+6^ weeks (OR: 13.142; 95% CI: 5.007–34.498; *p* < 0.001) (Table 4).

With regard to neonatal respiratory disorders, early gestational age at delivery, cesarean section, no labor and fetal distress were identified as independent risk factors; at 24^+0^–27^+6^ weeks, the risk for respiratory disorders was almost 77 times higher than at 32^+0^–36^+6^ weeks (OR: 77.271; 95% CI: 10.659–560.182; *p* < 0.001), and at 28^+0^–31^+6^ weeks, the risk was almost 5 times higher (OR: 5.802; 95% CI:3.682–9.144; *p* < 0.001). The cesarean delivery increased the risk for respiratory disorders by almost two times (OR: 2.208; 95% CI: 1.574–3.097; *p* < 0.001) in preterm neonates. Moreover, in cases with no onset of labor and fetal distress, an increased risk for respiratory disorders was noticed (OR: 1.789; 95% CI: 1.167–2.741; *p* = 0.008 and OR: 1.691; 95% CI: 1.060–2.699; *p* = 0.028, respectively) (Table 5). Multiple gestation was identified as the only independent risk factor for NEC (OR: 3.545; 95% CI: 1.207–10.411; *p* = 0.021) (Table 6). Furthermore, early gestational age at delivery was an independent risk factor for IVH; at 24–28 weeks, the risk for IVH was 112 times higher than at 32^+0^–36^+6^ weeks (OR: 112.783; 95% CI: 49.577–256.570; *p* < 0.001), and at 28^+0^–31^+6^ weeks, the risk was 20 times higher than at 32^+0^–36^+6^ weeks (OR: 20.200; 95% CI: 9.079–44.944; *p* < 0.001) (Table 7).

## 4. Discussion

In our cohort of preterm deliveries, we found that (i) the majority were moderate-to-late preterm neonates (32^+0^–36^+6^ weeks), (ii) after 28^+0^ weeks, most of the neonates were delivered via cesarean section, (iii) no labor, multiple gestation, non-cephalic presentation and fetal distress were independent risk factors of cesarean delivery, (iv) spontaneous onset of labor, multiple gestation and fetal distress were the main causes of prematurity at 32^+0^–36^+6^ weeks, (v) early gestational age at delivery was significantly associated with an increased risk of neonatal death, (vi) early gestational age at delivery, cesarean section, no labor and fetal distress were identified as independent risk factors for neonatal respiratory disorders, (vii) multiple gestation was associated with NEC and (viii) early gestational age at delivery was the only independent risk factor for IVH.

In our study, the rate of births before 32^+0^ weeks was 2.9%, and after 32^+0^ weeks, it was 4.6%. This is consistent with previous studies that reported that the preterm birth rate < 32 weeks is about 1.6%, while the rates of moderate-to-late prematurity varies from 4.2 to 8.9%, which is in accordance to our findings [19]. The relatively higher rate of deliveries < 32^+0^ weeks may be explained by the fact that our center acts as a referral tertiary center for several secondary units without a neonatal intensive care unit.

To date, the existing guidelines do not explicitly address the mode of delivery for preterm neonates [6]. A Cochrane review on preterm delivery in singletons with cephalic or breech presentation found insufficient evidence to suggest a standard policy for the mode of delivery [11]. As per our findings, the majority of preterm neonates were delivered via cesarean section, except those delivered extremely preterm (24^+0^–27^+6^ weeks), who were more likely to be born vaginally. This could be explained by the fact that neonatal mortality and morbidity are severely increased before 28 weeks, regardless of the mode of delivery. Consequently, considering that the risks of a cesarean may outweigh the benefits, vaginal delivery was preferred over a cesarean section in extremely preterm neonates. However, early preterm neonates were more likely to be born via cesarean section than the moderately late preterm ones. This could be attributed to an unfavorable bishop score or to the susceptibility to stress in relation to moderately late preterm neonates.

Regarding preterm neonates in breech presentation, non-cephalic presentation was one of the main causes of cesarean section in our sample; this is in accordance with findings from a systematic review, which found that neonatal mortality was lower in the group of neonates with breech presentation born via cesarean section (3.8%) compared to those born vaginally (11.5%) [20]. As for multiple gestations, data on the mode of delivery to be chosen are unclear; according to the current guidelines, in twin pregnancies, cesarean section should be performed when the first twin is in breech presentation [21]. The preferred mode of delivery in multiple pregnancies was cesarean section in our study, which was in agreement with the literature [22]. Regarding the mode of delivery in cases of medically indicated preterm births without onset of labor, a planned cesarean section seems to be the preferred method of delivery rather than induction of labor, according to the literature [23]. An unfavorable cervix, prolonged induction and fetal intolerance are probably the main reasons why cesarean section is preferred, which is in agreement with the results of our cohort.

One of the main causes of prematurity at 32^+0^ to 36^+6^ weeks of gestation was multiple gestation; the mean gestational age at delivery was 35 weeks for twin gestations and 32 weeks for triplets, according to two studies [24,25]. Moreover, fetuses less than 37 weeks old and with a birthweight of less than 2500 g are a high-risk group in terms of developing perinatal asphyxia; hence, emergency cesarean section was performed more often due to fetal distress among preterm pregnancies [26]. Spontaneous onset of labor was found to be more common after 32 weeks in our study; according to published data, women in their first pregnancy had the highest risk for a spontaneous preterm birth in the late preterm period [27].

As far as neonatal survival is concerned in the preterm period, the mode of delivery was not found to be a risk factor for neonatal mortality; this was in agreement with various studies in the literature [28,29]. Particularly, in a cohort study of very-low-birthweight neonates (<1500 g), there were no increased odds of death, severe IVH, NEC or sepsis in the vaginally delivered compared to the cesarean group [29]. It is supported by some data that preterm neonates exposed to the second stage of labor during a vaginal delivery have an increased risk of mild IVH [30], while another study correlated the incidence of IVH with the birthweight, the gestational age and the active phase of labor, regardless of the route of delivery [31].

With regard to neonatal respiratory disorders, we found that early gestational age at delivery, cesarean section and fetal distress were independent risk factors. This is in agreement with published data which found that neonates born via cesarean section were more likely to have respiratory distress syndrome (OR:1.79; 95% CI: 1.10–2.90), require intubation (OR: 1.80; 95% CI: 1.12–2.88) and have a longer stay at the neonatal intensive care unit (70.0 ± 37.1 vs. 57.3 ± 40.1 days; *p* = 0.02) [32]. Furthermore, it is supported that spontaneous preterm onset of preterm labor is associated with a decreased risk of respiratory disorders in comparison to induced preterm labor, which is also in accordance with our findings [14]. Published data have also shown that acute fetal distress, elective cesarean delivery, low Apgar score, prematurity, male gender and macrosomia are independent predictors of respiratory disorders [33].

NEC was significantly increased in multiple gestations; according to a population-based observational study, multiple gestations delivered via cesarean section were associated with higher odds for NEC (OR: 1.31) compared to singletons [34]. Moreover, NEC is reported to be more common in monochorionic twin gestations compared to dichorionic due to the presence of unbalanced inter-fetal transfusion through arterial–venous anastomoses in the placenta and ischemic damage caused by intra-uterine fetal hypotension or anemia [35].

Regarding IVH, early gestational age at delivery was the only risk factor found to be significant in our study. A study showed that neonates with IVH had a significantly lower gestational age (28.2 ± 2.6 vs. 30.4 ± 2.6 weeks; *p* < 0.003) [31]. The active phase of labor, regardless of the mode of delivery, is reported to increase the incidence of IVH [31], while most studies agree that the mode of delivery does not have any impact on the risk of IVH [29,31].

One of the major strengths of our study is the relatively large sample size. Furthermore, to our knowledge, this is the first study on neonatal outcomes of preterm deliveries in Greece. Our Academic Department serves a large population in Northern Greece; therefore, the results could probably be generalized to the entire Greek population and may contribute to a safer management of preterm gestations in the future. On the other hand, the study has certain limitations; its retrospective nature should be considered as the major one. However, there were no missing data because of the digital recording of all deliveries. Moreover, changes in local or national practice over the ten-year period may have affected the results, thus leading to bias. The latter could be eliminated by the fact that all the preterm neonates were managed by the same obstetrical department and neonatal intensive care unit; uniform medical protocols were used for all the cases, thus minimizing the risk of bias. Finally, although large, the sample size was still not adequate to avoid some wide confidence intervals in specific analyses.

Most preterm deliveries occurred in the moderate-to-late preterm period via cesarean section. Cesarean delivery was associated with no spontaneous onset of labor, multiple gestation, non-cephalic presentation and fetal distress, while early gestational age at delivery was considered a major prognostic factor of neonatal morbidity and mortality. The mode of delivery did not have any impact on neonatal survival, but premature neonates born via cesarean section were at higher risk of respiratory disorders than those born via vaginal delivery. To date, there is still conflicting and inconclusive evidence regarding the best mode of delivery of preterm neonates. More large longitudinal studies are necessary to establish definitive answers for each gestational age window and thus improve perinatal outcomes.

## Figures and Tables

**Table 1:** Mode of delivery according to gestational age.

Gestational Age	Mode of Delivery		Total	Multiple Logistic Regression		
	Vaginal Delivery	Cesarean Delivery		*p*-Value	ORs	95% CI
24^+0^–27^+6^ weeks	67 (52.8%)	60 (47.2%)	127	**<0.001**	**0.260**	**0.176–0.384**
28^+0^–31^+6^ weeks	51 (15.7%)	274 (84.3%)	325	**0.012**	**1.561**	**1.104–2.208**
32^+0^–36^+6^ weeks (Reference)	161 (22.5%)	554 (77.5%)	715	Reference		
Total	279 (23.9%)	888 (76.1%)	1.167			

Reference: vaginal delivery, ORs: odds ratios, CI: confidence interval. Statistically significant results are highlighted in bold.

**Table 2:** Mode of delivery according to causes of preterm deliveries.

Causes of Preterm Delivery	Mode of Delivery		Univariate Analysis			Multivariate Analysis		
	Vaginal Delivery	Cesarean Delivery	*p*-Value	OR	95% CI	*p*-Value	OR	95% CI
Onset of labor—spontaneous (Reference)	275 (47.7%)	302 (52.3%)						
Onset of labor—no labor	4 (0.7%)	586 (99.3%)	**<0.001**	**133.402**	**49.233–361.466**	**<0.001**	**75.440**	**16.654–341.731**
PPROM (Reference)	166 (75.8%)	53 (23.9%)						
No amniotic fluid disorder	722 (76.2%)	226 (23.8%)	0.910	1.020	0.724–1.438	0.313	1.297	0.782–2.149
No preeclampsia/ HELLP (Reference)	277 (26.7%)	762 (73.3)						
Preeclampsia/ HELLP	2 (1.6%)	126 (98.4%)	**<0.001**	**22.902**	**5.627–93.207**	0.546	1.833	0.256–13.118
No FGR (Reference)	276 (28.3%)	699 (71.1%)						
FGR	2 (1%)	189 (99%)	**<0.001**	**37.313**	**9.200–151.338**	0.606	1.673	0.237–11.795
Singleton gestations (Reference)	252 (31.8%)	541 (68.2%)						
Multiple gestations	27 (7.2%)	347 (92.8%)	**<0.001**	**5.986**	**3.937–9.103**	**<0.001**	**11.002**	**6.806–17.785**
Cephalic fetal presentation (Reference)	278 (26.2%)	785 (73.8%)						
Non-cephalic fetal presentation	1 (1%)	103 (99%)	**<0.001**	**36.476**	**5.066–262.662**	**<0.001**	**137.230**	**18.694–1.007.394**
No fetal distress (Reference)	272 (30.2%)	629 (69.8%)						
Fetal distress	7 (2.6%)	258 (97.4%)	**<0.001**	**15.938**	**7.423–34.221**	**<0.001**	**8.178**	**3.398–19.686**

PPROM: preterm prelabor rupture of membranes, HELLP: hemolysis, elevated liver enzymes and low platelets, FGR: fetal growth restriction, OR: odds ratio, CI: confidence interval. Statistically significant results are highlighted in bold.

**Table 3:** Causes of preterm deliveries according to gestational age.

Gestational Age	Causes of Preterm Delivery	Multivariate Analysis		
		*p*-Value	OR	95% CI
32^+0^–36^+6^ weeks	Spontaneous onset of labor	**<0.001**	**6.038**	**3.163–11.527**
	Amniotic fluid disorder	0.560	1.182	0.674–2.071
	Preeclampsia/ HELLP	0.117	2.148	0.826–5.589
	FGR	0.124	0.422	0.140–1.267
	Multiple gestation	**0.008**	**1.782**	**1.165–2.227**
	Non-cephalic fetal presentation	0.148	1.647	0.838–3.240
	Fetal distress	**<0.001**	**5.326**	**2.796–10.144**
28^+0^–31^+6^ weeks	Spontaneous onset of labor	0.393	1.335	0.688–2.591
	Amniotic fluid disorder	0.061	0.569	0.315–1.027
	Preeclampsia/ HELLP	0.183	0.508	0.187–1.377
	FGR	**<0.001**	**0.142**	**0.047–0.430**
	Multiple gestation	0.585	1.135	0.720–1.789
	Non-cephalic fetal presentation	0.852	0.933	0.447–1.946
	Fetal distress	0.199	0.661	0.351–1.244
24^+0^–27^+6^ weeks	Reference			

HELLP: hemolysis, elevated liver enzymes and low platelets, FGR: fetal growth restriction, OR: odds ratio, CI: confidence interval. Statistically significant results are highlighted in bold.

**Table 4:** Association of gestational age and mode of delivery with neonatal survival.

Gestational Age (Weeks)	Neonatal Outcome		Multivariate Analysis		
	Death	Survival	*p*-Value	ORs	95% CI
24^+0^–27^+6^ weeks	63 (49.6%)	64 (50.4%)	**<0.001**	**129.493**	**49.761–336.979**
28^+0^–31^+6^ weeks	27 (8.3%)	298 (91.7%)	**<0.001**	**13.142**	**5.007–34.498**
32^+0^–36^+6^ weeks	5 (0.7%)	710 (99.3%)	Reference		
Mode of delivery					
Cesarean delivery	53 (6%)	835 (94%)	0.328	1.317	0.759–2.284
Vaginal delivery	42 (15.1%)	237 (84.9%)	Reference		

Statistically significant results are highlighted in bold.

**Table 5:** Respiratory disorders’ risk according to gestational age, mode of delivery and different causes of preterm delivery.

Gestational Age (Weeks)	Neonatal Complications		Multivariate Analysis		
	RDS	Without RDS	*p*-Value	ORs	95% CI
24^+0^–27^+6^ weeks	126 (99.2%)	1 (0.8%)	**<0.001**	**77.271**	**10.659–560.182**
28^+0^–31^+6^ weeks	302 (92.9%)	23 (7.1%)	**<0.001**	**5.802**	**3.682–9.144**
32^+0^–36^+6^ weeks	491 (68.7%)	224 (31.3%)	Reference		
Mode of delivery					
Cesarean delivery	720 (81.1%)	168 (18.9%)	**<0.001**	**2.208**	**1.574–3.097**
Vaginal delivery	199 (71.3%)	80 (28.7%)	Reference		
Causes of preterm birth					
No labor	465 (78.8%)	125 (21.2%)	**0.008**	**1.789**	**1.167–2.741**
PPROM	164 (74.9%)	55 (25.1%)	---	---	---
Preeclampsia/ HELLP	105 (82%)	23 (18%)	---	---	---
FGR	147 (77%)	44 (23%)	---	---	---
Multiple gestation	307 (82.1%)	67 (17.9%)	---	---	---
Non-cephalic presentation	82 (78.8%)	22 (21.2%)	---	---	---
Fetal distress	231 (87.2%)	34 (12.8%)	**0.028**	**1.691**	**1.060–2.699**

PPROM: preterm prelabor rupture of membranes, HELLP: hemolysis, elevated liver enzymes and low platelets, FGR: fetal growth restriction, ORs: odds ratios, CI: confidence interval. Statistically significant results are highlighted in bold.

**Table 6:** Necrotizing enterocolitis’ risk according to gestational age, mode of delivery and different causes of preterm delivery.

Gestational Age (Weeks)	Neonatal Complications		Multivariate Analysis		
	NEC	Without NEC	*p*-Value	ORs	95% CI
24^+0^–27^+6^ weeks	13 (10.2%)	114 (89.8%)	0.142	0.539	0.237–1.229
28^+0^–31^+6^ weeks	17 (5.2%)	308 (94.8%)	0.990	0.000	0.001–1.001
32^+0^–36^+6^ weeks	0 (0%)	715 (100%)	Reference		
Mode of delivery					
Cesarean delivery	19 (2.1%)	869 (97.9%)	0.512	0.753	0.322–1.759
Vaginal delivery	11 (3.9%)	268 (96.1%)	Reference		
Causes of preterm birth					
No labor	13 (2.2%)	577 (97.8%)	---	---	---
PPROM	6 (2.7%)	213 (97.3%)	---	---	---
Preeclampsia/ HELLP	3 (2.3%)	125 (97.7%)	---	---	---
FGR	1 (0.5%)	190 (99.5%)	---	---	---
Multiple gestation	4 (1.1%)	370 (98.9%)	**0.021**	**3.545**	**1.207–10.411**
Non-cephalic presentation	2 (1.9%)	102 (98.1%)	---	---	---
Fetal distress	11 (4.2%)	254 (95.8%)	---	---	---

NEC: necrotizing enterocolitis, PPROM: preterm prelabor rupture of membranes, HELLP: hemolysis, elevated liver enzymes and low platelets, FGR: fetal growth restriction, ORs: odds ratios, CI: confidence interval. Statistically significant results are highlighted in bold.

**Table 7:** Intraventricular hemorrhage risk according to gestational age, mode of delivery and different causes of preterm delivery.

Gestational Age (Weeks)	Neonatal Complications		Multivariate Analysis		
	IVH	Without IVH	*p*-Value	ORs	95% CI
24^+0^–27^+6^ weeks	67 (52.8%)	60 (47.2%)	**<0.001**	**112.783**	**49.577–256.570**
28^+0^–31^+6^ weeks	55 (16.9%)	270 (83.1%)	**<0.001**	**20.200**	**9.079–44.944**
32^+0^–36^+6^ weeks	7 (1%)	708 (99%)	Reference		
Mode of delivery					
Cesarean delivery	83 (9.3%)	805 (90.7%)	0.798	1.091	0.561–2.124
Vaginal delivery	46 (16.5%)	233 (83.5%)	Reference		
Causes of preterm birth					
No labor	55 (9.3%)	535 (90.7%)	---	---	---
PPROM	18 (8.2%)	201 (91.8%)	---	---	---
Preeclampsia/ HELLP	10 (7.8%)	118 (92.2%)	---	---	---
FGR	16 (8.4%)	175 (91.6%)	---	---	---
Multiple gestation	37 (9.9%)	337 (90.1%)	---	---	---
Abnormal presentation	11 (10.6%)	93 (89.4%)	---	---	---
Fetal distress	38 (14.3%)	227 (85.7%)	---	---	---

IVH: intraventricular hemorrhage, PPROM: preterm prelabor rupture of membranes, HELLP: hemolysis, elevated liver enzymes and low platelets, FGR: fetal growth restriction, ORs: odds ratios, CI: confidence interval. Statistically significant results are highlighted in bold.

## Data Availability

Data are available upon request.

## Supplementary Materials

The following supporting information can be downloaded at: https://www.mdpi.com/article/10.3390/medicina60010010/s1, Table S1: Comparison of the mode of delivery in different gestational ages according to onset of labor (spontaneous/no labor); Table S2: Comparison of the mode of delivery in different gestational ages according to amniotic fluid (PPROM/normal amniotic fluid); Table S3: Comparison of the mode of delivery in different gestational ages according to the presence of preeclampsia (preeclampsia/HELLP vs. no preeclampsia/HELLP); Table S4: Comparison of the mode of delivery in different gestational ages according to fetal growth (FGR/normal growth); Table S5: Comparison of the mode of delivery in different gestational ages according to number of fetuses (multiple/singleton pregnancies); Table S6: Comparison of the mode of delivery in different gestational ages according to presentation (non-cephalic/cephalic presentation); Table S7: Comparison of the mode of delivery in different gestational ages according to fetal status (fetal distress/no fetal distress).

## References

[1] Muglia L.J., Katz M. (2010). The enigma of spontaneous preterm birth. N. Engl. J. Med..

[2] Romero R., Dey S.K., Fisher S.J. (2014). Preterm labor: One syndrome, many causes. Science.

[3] Rubens C.E., Sadovsky Y., Muglia L., Gravett M.G., Lackritz E., Gravett C. (2014). Prevention of preterm birth: Harnessing science to address the global epidemic. Sci. Transl. Med..

[4] Englund-Ogge L., Brantsaeter A.L., Haugen M., Sengpiel V., Khatibi A., Myhre R., Myking S., Meltzer H.M., Kacerovsky M., Nilsen R.M. (2012). Association between intake of artificially sweetened and sugar-sweetened beverages and preterm delivery: A large prospective cohort study. Am. J. Clin. Nutr..

[5] Englund-Ögge L., Brantsaeter A.L., Sengpiel V., Haugen M., Birgisdottir B.E., Myhre R., Meltzer H.M., Jacobsson B. (2014). Maternal dietary patterns and preterm delivery: Results from large prospective cohort study. BMJ.

[6] Giouleka S.M., Tsakiridis I., Kostakis N., Koutsouki G., Kalogiannidis I., Mamopoulos A., Athanasiadis A., Dagklis T. (2022). Preterm Labor: A Comprehensive Review of Guidelines on Diagnosis, Management, Prediction and Prevention. Obstet. Gynecol. Surv..

[7] Dagklis T., Tsakiridis I., Mamopoulos A., Dardavessis T., Athanasiadis A. (2020). Modifiable risk factors for spontaneous preterm birth in nulliparous women: A prospective study. J. Perinat. Med..

[8] Di Renzo G.C., Giardina I., Rosati A., Clerici G., Torricelli M., Petraglia F., The Italian Preterm Network Study Group (2011). Maternal risk factors for preterm birth: A country-based population analysis. Eur. J. Obstet. Gynecol. Reprod. Biol..

[9] Morken N.H., Kallen K., Jacobsson B. (2014). Predicting risk of spontaneous preterm delivery in women with a singleton pregnancy. Paediatr. Perinat. Epidemiol..

[10] Grant A., Glazener C.M. (2001). Elective versus selective caesarean section for delivery of the small baby. Cochrane Database Syst. Rev..

[11] Alfirevic Z., Milan S.J., Livio S. (2013). Caesarean section versus vaginal delivery for preterm birth in singletons. Cochrane Database Syst. Rev..

[12] Grant A., Penn Z.J., Steer P.J. (1996). Elective or selective caesarean delivery of the small baby? A systematic review of the controlled trials. Br. J. Obstet. Gynaecol..

[13] Mercer B.M. (2013). Mode of delivery for periviable birth. Semin. Perinatol..

[14] Wylie B.J., Davidson L.L., Batra M., Reed S.D. (2008). Method of delivery and neonatal outcome in very low-birthweight vertex-presenting fetuses. Am. J. Obstet. Gynecol..

[15] Furukawa S., Sameshima H., Ikenoue T. (2014). The impact of cesarean section on neonatal outcome of infants born at 23 weeks of gestation. Early Hum. Dev..

[16] Malloy M.H., Doshi S. (2008). Cesarean section and the outcome of very preterm and very low-birthweight infants. Clin. Perinatol..

[17] Tsakiridis I., Mamopoulos A., Athanasiadis A., Dagklis T. (2020). Antenatal Corticosteroids and Magnesium Sulfate for Improved Preterm Neonatal Outcomes: A Review of Guidelines. Obstet. Gynecol. Surv..

[18] Wade D.T. (2005). Ethics, audit, and research: All shades of grey. BMJ.

[19] Tsakiridis I., Dagklis T., Sotiriadis A., Mamopoulos A., Zepiridis L., Athanasiadis A. (2023). Third-trimester cervical length assessment for the prediction of spontaneous late preterm birth. J. Matern. Fetal Neonatal Med..

[20] Bergenhenegouwen L., Meertens L., Schaaf J., Nijhuis J., Mol B., Kok M., Scheepers H. (2014). Vaginal delivery versus caesarean section in preterm breech delivery: A systematic review. Eur. J. Obstet. Gynecol. Reprod. Biol..

[21] Tsakiridis I., Giouleka S., Mamopoulos A., Athanasiadis A., Dagklis T. (2020). Management of Twin Pregnancies: A Comparative Review of National and International Guidelines. Obstet. Gynecol. Surv..

[22] Barzilay E., Mazaki-Tovi S., Amikam U., de Castro H., Haas J., Mazkereth R., Sivan E., Schiff E., Yinon Y. (2015). Mode of delivery of twin gestation with very low birthweight: Is vaginal delivery safe?. Am. J. Obstet. Gynecol..

[23] Zhang Y., Zhou J., Ma Y., Liu L., Xia Q., Fan D., Ai W. (2019). Mode of delivery and preterm birth in subsequent births: A systematic review and meta-analysis. PLoS ONE.

[24] Fergal D.M. (2009). Multiple Gestation: Clinical Characteristics and Management. Creasy and Resnik’s Maternal-Fetal Medicine.

[25] Petrova I., Nikolov A., Markov P., Slancheva B., Yarakova N. (2013). Gestational age of delivery in multiple gestation. Akush Ginekol..

[26] Litorp H., Gurung R., Malqvist M., Kc A. (2020). Disclosing suboptimal indications for emergency caesarean sections due to fetal distress and prolonged labor: A multicenter cross-sectional study at 12 public hospitals in Nepal. Reprod. Health.

[27] Koullali B., van Zijl M.D., Kazemier B.M., Oudijk M.A., Mol B.W.J., Pajkrt E., Ravelli A.C.J. (2020). The association between parity and spontaneous preterm birth: A population based study. BMC Pregnancy Childbirth.

[28] Werner E.F., Han C.S., Savitz D.A., Goldshore M., Lipkind H.S. (2013). Health outcomes for vaginal compared with cesarean delivery of appropriately grown preterm neonates. Obstet. Gynecol..

[29] Durie D.E., Sciscione A.C., Hoffman M.K., Mackley A.B., Paul D.A. (2011). Mode of delivery and outcomes in very low-birth-weight infants in the vertex presentation. Am. J. Perinatol..

[30] Gawade P.L., Whitcomb B.W., Chasan-Taber L., Pekow P.S., Ronnenberg A.G., Shah B., Plevyak M.P., Markenson G.R. (2013). Second stage of labor and intraventricular hemorrhage in early preterm infants in the vertex presentation. J. Matern. Fetal Neonatal Med..

[31] Anderson G.D., Bada H.S., Sibai B.M., Harvey C., Korones S.B., Magill H.L., Wong S.P., Tullis K. (1988). The relationship between labor and route of delivery in the preterm infant. Am. J. Obstet. Gynecol..

[32] Van Winden K.R., Pathak B., Barton L., Opper N., Lane C.J., Ramanathan R., Ouzounian J.G., Lee R.H., Blue N.R. (2015). Neonatal Outcomes by Mode of Delivery in Preterm Birth. Am. J. Perinatol..

[33] Tochie J.N., Choukem S.P., Langmia R.N., Barla E., Koki-Ndombo P. (2016). Neonatal respiratory distress in a reference neonatal unit in Cameroon: An analysis of prevalence, predictors, etiologies and outcomes. Pan Afr. Med. J..

[34] Riskin A., Riskin-Mashiah S., Itzchaki O., Bader D., Zaslavsky-Paltiel I., Lerner-Geva L., Reichman B. (2021). Mode of delivery and necrotizing enterocolitis in very preterm very-low-birth-weight infants. J. Matern. Fetal Neonatal Med..

[35] Aapkes R., Hack K., Koopman-Esseboom C., Nikkels P., Derks J., Brouwers H. (2019). Necrotizing Enterocolitis in Multi Fetal Pregnancies: Can We Find a Key in Placental Abnormalities? A Retrospective Data Analysis. Open J. Obstet. Gynecol..

