# Supplementary material for: Mode of Delivery and Neonatal Outcomes of Preterm Deliveries: A Retrospective Study in Greece
Source: Medicina (Kaunas). 2023 Dec 20;60(1):10. doi: 10.3390/medicina60010010 (PMC10820495; doi:10.3390/medicina60010010)
Supplement: Supplementary file 1 [file medicina-60-00010-s001.zip › medicina-2710784-supplementary.pdf]

medicina MDPI

Supplementary Table S1. Comparison of the mode of delivery in different gestational ages according to onset of labor (spontaneous/no labor).

**Gestational Age**	**Mode of Delivery in pregnancies with no onset labor**		**Univariate Analysis**		
	**Cesarean Delivery**	**Vaginal Delivery**	***p-value***	**ORs**	**95% CI**
24^{+0}-27^{+6} weeks	34 (97.1%)	1 (2.9%)	**<0.001**	**86.308**	**11.226-663.563**
28^{+0}-31^{+6} weeks	170 (100%)	0 (0%)	**<0.001**	**168.052**	**11.120-590.980**
32^{+0}-36^{+6} weeks	382 (99.2%)	3 (0.8%)	**<0.001**	**116.969**	**36.802-371.771**

Reference: spontaneous onset of labor

Supplementary Table S2. Comparison of the mode of delivery in different gestational ages according to amniotic fluid (PPROM/normal amniotic fluid).

**Gestational Age**	**Mode of Delivery in PPROM**		**Univariate Analysis**		
	**Cesarean Delivery**	**Vaginal Delivery**	***p-value***	**ORs**	**95% CI**
24^{+0}-27^{+6} weeks	14 (70%)	6 (30%)	**0.032**	**3.094**	**1.105-8.668**
28^{+0}-31^{+6} weeks	51 (83.6%)	10 (16.4%)	0.867	1.066	0.501-2.270
32^{+0}-36^{+6} weeks	101 (73.2%)	37 (26.8%)	0.179	1.338	0.874-2.049

Reference: normal fluid

PPROM: preterm prelabor rupture of membranes

Supplementary Table S3. Comparison of the mode of delivery in different gestational ages according to the presence of preeclampsia/HELLP (preeclampsia/HELLP vs no preeclampsia/HELLP).

**Gestational Age**	**Mode of Delivery in pregnancies with preeclampsia/HELLP**		**Univariate Analysis**		
	**Cesarean Delivery**	**Vaginal Delivery**	***p-value***	**ORs**	**95% CI**
24^{+0}-27^{+6} weeks	8 (100%)	0 (0%)	**0.016**	**9.019**	**1.076-75.621**
28^{+0}-31^{+6} weeks	36 (100%)	0 (0%)	**0.022**	**7.500**	**1.004-56.011**
32^{+0}-36^{+6} weeks	82 (97.6%)	2 (2.4%)	**<0.001**	**13.811**	**3.358-56.807**

Reference: no preeclampsia/HELLP (hemolysis, elevated liver enzymes, low platelets)

Supplementary Table S4. Comparison of the mode of delivery in different gestational ages according to fetal growth (FGR/normal growth).

**Gestational Age**	**Mode of Delivery in FGR**		**Univariate Analysis**		
	**Cesarean Delivery**	**Vaginal Delivery**	***p*-value**	**ORs**	**95% CI**
24^{+0}-27^{+6} weeks	3 (75%)	1 (25%)	0.259	3.474	0.352-34.328
28^{+0}-31^{+6} weeks	71 (100%)	0 (0%)	**<0.001**	**17.241**	**2.338-127.146**
32^{+0}-36^{+6} weeks	115 (99.1%)	1 (0.9%)	**<0.001**	**41.913**	**5.806-302.590**

Reference: normal growth

FGR: fetal growth restriction

Supplementary Table S5. Comparison of the mode of delivery in different gestational ages according to number of fetuses (multiple/singleton pregnancies).

**Gestational Age**	**Mode of Delivery in multiple pregnancies**		**Univariate Analysis**		
	**Cesarean Delivery**	**Vaginal Delivery**	***p-value***	**ORs**	**95% CI**
24^{+0}-27^{+6} weeks	19 (42.2%)	26 (57.8%)	0.401	0.731	0.351-1.521
28^{+0}-31^{+6} weeks	104 (100%)	0 (0%)	**<0.001**	**30.900**	**4.206-226.992**
32^{+0}-36^{+6} weeks	224 (99.6%)	1 (0.4%)	**<0.001**	**108.606**	**15.095-781.421**

Reference: singleton pregnancy

Supplementary Table S6. Comparison of the mode of delivery in different gestational ages according to fetal presentation (non-cephalic/cephalic presentation).

**Gestational Age**	**Mode of Delivery in non-cephalic presentation**		**Univariate Analysis**		
	**Cesarean Delivery**	**Vaginal Delivery**	***p*-*value***	**ORs**	**95% CI**
24^{+0}-27^{+6} weeks	12 (92.3%)	1 (7.7%)	**<0.001**	**16.500**	**2.075-131.231**
28^{+0}-31^{+6} weeks	27 (100%)	0 (0%)	**0.019**	**5.368**	**0.712-40.466**
32^{+0}-36^{+6} weeks	64 (100%)	0 (0%)	**<0.001**	**20.700**	**2.848-150.445**

Reference: cephalic presentation

Supplementary Table S7. Comparison of the mode of delivery in different gestational ages according to fetal status (fetal distress/no fetal distress).

**Gestational Age**	**Mode of Delivery in pregnancies with fetal distress**		**Univariate Analysis**		
	**Cesarean Delivery**	**Vaginal Delivery**	***p*-*value***	**ORs**	**95% CI**
24^{+0}-27^{+6} weeks	23 (79.3%)	6 (20.7%)	**<0.001**	**6.320**	**2.355-16.956**
28^{+0}-31^{+6} weeks	120 (100%)	0 (0%)	**<0.001**	**39.409**	**5.368-289.307**
32^{+0}-36^{+6} weeks	115 (99.1%)	1 (0.9%)	**<0.001**	**42.009**	**5.819-303.282**

Reference: no fetal distress
